# Endoscopic rescue management of stent displacement after a pancreatic pseudocyst endoscopic drainage

**DOI:** 10.1055/a-2589-1716

**Published:** 2025-05-14

**Authors:** Yue Hu, Bin Lu, Yi Xu, Liang Huang

**Affiliations:** 1 74723 Gastroenterology, The First Affiliated Hospital of Zhejiang Chinese Medical University, Zhejiang Provincial Hospital of Chinese Medicine, Zhejiang, China; 2 74723 Key Laboratory of Digestive Pathophysiology of Zhejiang Province, The First Affiliated Hospital of Zhejiang Chinese Medical University, Zhejiang, China

A 55-year-old woman had a history of acute pancreatitis 4 years prior, with the development of a large pancreatic pseudocyst (62 mm × 77 mm × 72mm) in the pancreatic tail ( Fig. 1 ). Endoscopic ultrasound (EUS)-guided pseudocyst drainage was successfully performed with a 10 Fr plastic double-pigtail stent. Post-procedural follow-up computed tomography (CT) imaging at 1 month revealed persistent pseudocyst dimensions with stent migration into the cystic cavity ( Fig. 2 ). After obtaining informed consent, the patient was referred for re-endoscopic pseudocyst drainage.

**Fig. 1:**
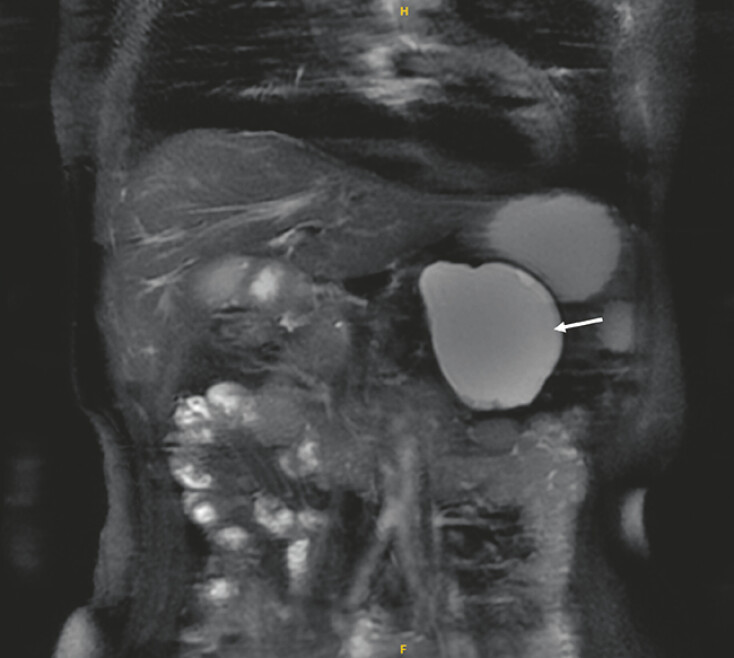
Magnetic resonance imaging scan showing a pancreatic pseudocyst (arrow) located in the tail of the pancreas.

**Fig. 2:**
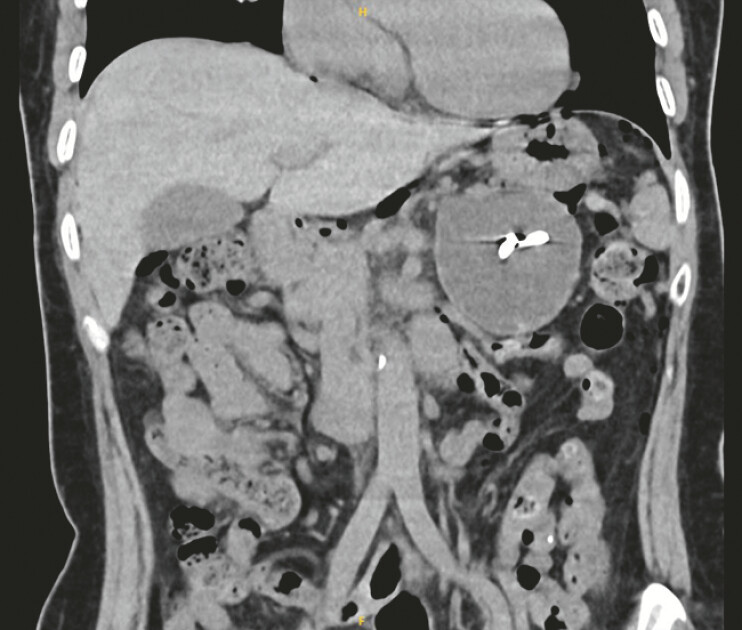
Abdominal computed tomography scan view showing complete migration of the first stent into the pseudocyst cavity.

However, we did not observe any small orifice at the location of the previously applied stent during the endoscopy. EUS images confirmed stent migration into the pseudocyst lumen. We proceeded with transgastric puncture of the pseudocyst using a 19-gauge fine-needle aspiration needle under EUS guidance. A 0.035-inch guidewire was advanced through the needle, and the tract was dilated to 10 mm ( Fig. 3 **a** ). After entering the pseudocyst lumen, the intracystic migrated stents were then removed by using foreign body forceps ( Fig. 3 **b** ). Surprisingly, we found a full-layer perforation of the fistulous tract following balloon dilation ( Fig. 3 **c** ). To simultaneously address perforation and cyst drainage, we placed a fully covered metal stent through the cystogastrostomy, fixing the proximal end on the stomach side with metal clips ( Fig. 4 and Video 1 ). Effective drainage of the pseudocyst was observed, and the patient remained well and was discharged after 6 days. At follow-up 3 months later, an abdominal CT scan showed complete resolution of the pseudocyst, the stent was removed endoscopically ( Fig. 5 ).

**Fig. 3:**
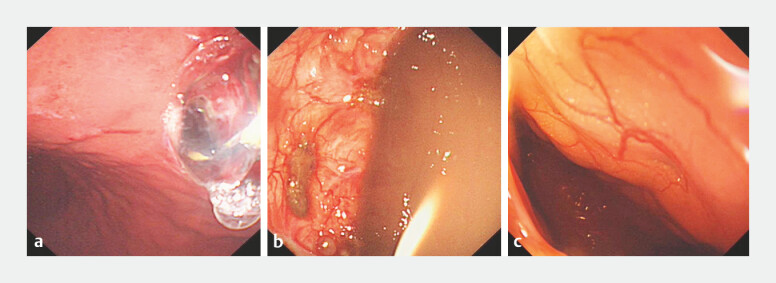
Endoscopic view showing balloon dilation to re-establishment of the gastro-pancreatic tunnel. **a** endoscopic balloon dilation; **b** pseudocyst lumen; **c** abdominal cavity.

**Fig. 4:**
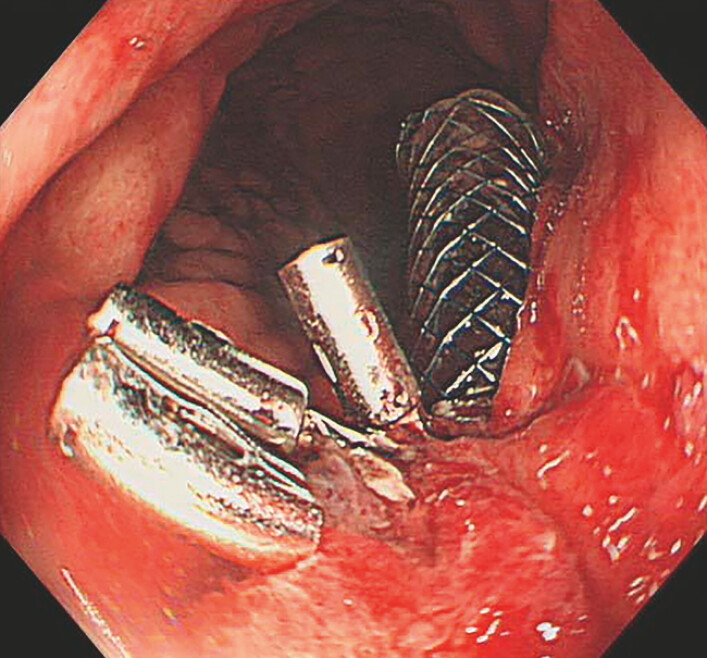
Endoscopic view showing the final stent position.

Endoscopic rescue management of stent displacement after a pancreatic pseudocyst endoscopic drainage.Video 1

**Fig. 5:**
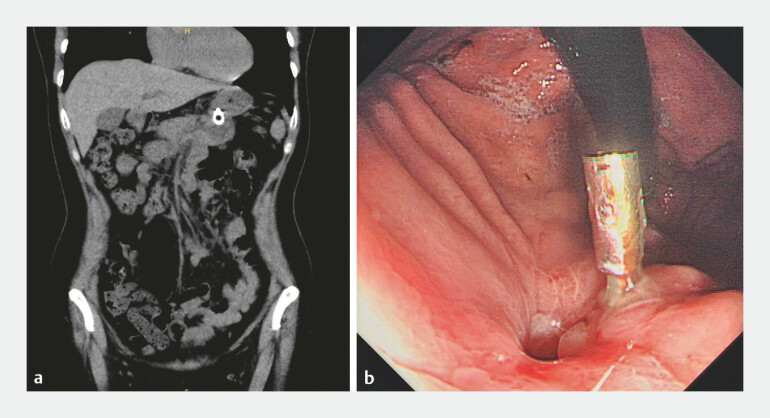
Images showing the follow-up of the pancreatic pseudocyst. **a** abdominal computed tomography scan; **b** endoscopy.

Stent migration represents a common complication in the endoscopic management of pancreatic pseudocysts 1 , with endoscopic salvage procedures offering viable therapeutic options 2 3 . This case highlights the potential risk of perforation during endoscopic re-establishment of the gastro-pancreatic tunnel. The deployment of covered stents demonstrates efficacy in drainage and perforation closure, sparing patients from the morbidity associated with surgical intervention 4 .

Endoscopy_UCTN_Code_TTT_1AO_2AO

## Notes

Endoscopy E-Videos https://eref.thieme.de/e-videos
*E-Videos* is an open access online section of the journal *Endoscopy* , reporting on interesting cases and new techniques in gastroenterological endoscopy. All papers include a high-quality video and are published with a Creative Commons CC-BY license. Endoscopy E-Videos qualify for HINARI discounts and waivers and eligibility is automatically checked during the submission process. We grant 100% waivers to articles whose corresponding authors are based in Group A countries and 50% waivers to those who are based in Group B countries as classified by Research4Life (see: https://www.research4life.org/access/eligibility/ ). This section has its own submission website at https://mc.manuscriptcentral.com/e-videos .
